# Development and Validation of Novel Free Vitamin D Equations: The Health Aging and Body Composition Study

**DOI:** 10.1002/jbm4.10781

**Published:** 2023-06-13

**Authors:** Jonathan H. Cheng, Andrew N. Hoofnagle, Ronit Katz, Stephen B. Kritchevsky, Michael G. Shlipak, Mark J. Sarnak, Joachim H. Ix, Charles Ginsberg

**Affiliations:** ^1^ Division of Nephrology‐Hypertension University of California San Diego CA USA; ^2^ Nephrology Section Veterans Affairs San Diego Healthcare System San Diego CA USA; ^3^ Department of Laboratory Medicine and Medicine and the Kidney Research Institute University of Washington Seattle WA USA; ^4^ Department of Obstetrics and Gynecology University of Washington Seattle WA USA; ^5^ Department of Internal Medicine, Section on Gerontology and Geriatric Medicine Wake Forest School of Medicine Winston‐Salem NC USA; ^6^ Kidney Health Research Collaborative, Veterans Affairs Medical Center University of California San Francisco CA USA; ^7^ Department of Medicine, Division of Nephrology Tufts Medical Center Boston MA USA

**Keywords:** FREE VITAMIN D, MINERAL METABOLISM, PTH/VIT D/FGF23, STATISTICAL METHODS, VITAMIN D BINDING PROTEIN

## Abstract

Vitamin D deficiency is prevalent in 25% of Americans. However, 25(OH)D may not be an accurate measure of vitamin D because the majority (85%–90%) of 25(OH)D is bound to vitamin D binding protein (VDBP), which varies by over 30% across individuals. Free 25(OH)D may be a better measure, but it is difficult to measure accurately and precisely. The existing free 25(OH)D estimating equation does not include VDBP phenotypes; therefore, new equations that include this variable may be more accurate. A total of 370 participants in the Health, Aging, and Body Composition Study, a cohort of healthy community‐dwelling individuals aged 70–79 years old, underwent VDBP and vitamin D metabolite [25(OH)D, 24,25(OH)_2_D, 1,25(OH)_2_D, free 25(OH)D] measurements and were randomly allocated into equation development (two out of three) and internal validation (one out of three) groups. New equations were developed with multiple linear regression and were internally validated with Bland–Altman plots. The mean age was 75 ± 3 years, 53% were female, and the mean measured free 25(OH)D was 5.37 ± 1.81 pg/mL. Three equations were developed. The first equation included albumin, 25(OH)D_3_, 25(OH)D_2_, VDBP, 1,25(OH)_2_D_3_, and 24,25(OH)_2_D_3_. The second equation included all variables in Eq. (1) plus VDBP phenotypes. The third equation included albumin, 25(OH)D_3_, intact parathyroid hormone, and 1,25(OH)_2_D_3_. In internal validation, all three new equations predicted free 25(OH)D values within 30% and 15% of the measured free 25(OH)D concentrations in 76%–80% and 48%–52% of study participants, respectively. Equation (2) was the most precise, with a mean bias of 0.06 (95% limits of agreement −2.41 to 2.30) pg/mL. The existing equation estimated free 25(OH)D within 30% and 15% of measured free 25(OH)D in 43% and 22% of participants, respectively. Free 25(OH)D can be estimated with clinically available biomarkers as well as with more laboratory‐intensive biomarkers with moderate precision. © 2023 The Authors. *JBMR Plus* published by Wiley Periodicals LLC on behalf of American Society for Bone and Mineral Research.

## Introduction

Vitamin D deficiency is believed to contribute to numerous adverse health outcomes, including mineral‐bone and cardiovascular diseases.^[^ ^1^, ^2^ ^]^ The current definition of vitamin D deficiency is 25‐hydroxyvitamin D [25(OH)D] <12 ng/mL according to the National Academies of Sciences, Engineering, and Medicine (NASEM), and many current guidelines recommend targeting 25(OH)D concentrations >20 ng/mL.^[^ ^3^, ^4^ ^]^ An estimated one in five Americans take vitamin D supplementation, yet questions remain about the measurement and interpretation of vitamin D concentrations.^[^ ^5^ ^]^ The current standard clinical biomarker of vitamin D status, 25(OH)D, may not be reliable in determining important clinical outcomes.^[^ ^6^, ^7^ ^]^ Thus, identification of biomarkers of vitamin D status that are accurate and predictive of clinical outcomes may facilitate identifying which patients may need supplementation and may ultimately lead to improved clinical outcomes. This clinical need is particularly pressing among older adults, given their higher absolute risk for adverse health outcomes, including bone and cardiovascular diseases.

Several studies have suggested that 25(OH)D may not accurately reflect biologically active vitamin D status due to variations in vitamin D binding protein (VDBP).^[^ ^7^, ^8^, ^9^, ^10^, ^11^ ^]^ 25(OH)D exists as either free (~1%) or as a protein‐bound moiety (~99% to VDBP with a small fraction to albumin).^[^ ^11^ ^]^ Previous studies showed substantial variability in VDBP concentrations and found multiple common genetic variants in VDBP that might lead to different binding affinities for 25(OH)D.^[^ ^8^, ^9^, ^10^, ^11^ ^]^ Therefore, concentrations of free 25(OH)D, which could be a more clinically relevant measure, may vary between individuals depending on VDBP concentrations and phenotype at any given measured level of 25(OH)D. However, free 25(OH)D is not commercially available, so an accurate equation to estimate free 25(OH)D may be beneficial for both clinical and research purposes.

To our knowledge, two prior teams of investigators have developed equations to estimate free 25(OH)D. The first was developed by Bikle et al. and included albumin, albumin binding affinity, VDBP, and VDBP binding affinity. The second equation used to estimate free 25(OH)D was modified from an equation used to estimate free testosterone concentrations and was developed by Vermulean et al.^[^ ^12^, ^13^, ^14^, ^15^ ^]^ The Vermulean equation included albumin, albumin binding affininity, VDBP, and VDBP binding affinity. Schwartz et. al found that these two equations produced almost identical free 25(OH)D estimates in 155 participants, which included two patient populations with reduced albumin, pregnant women (second and third trimesters), and people with liver disease (*r* ^2^ = 1.0, *p* < 0.0001).^[^ ^16^ ^]^ Neither of these equations included VDBP genetic phenotypes, which may have different binding affinities with vitamin D metabolites.^[^ ^14^, ^15^ ^]^ Several studies have investigated the relation between directly measured free 25(OH)D and calculated free 25(OH)D using these two equations.^[^ ^12^, ^16^, ^17^, ^18^ ^]^

Because these studies did not include either VDBP phenotypes, we hypothesized that these prior equations might have room for improvement. Thus, we sought to develop new equations for free 25(OH)D among participants in the Health, Aging, and Body Composition (Health ABC) study that incorporate important parameters, including VDBP concentration and VDBP phenotype.^[^ ^6^, ^8^, ^19^, ^20^ ^]^ We hypothesized that new free 25(OH)D equations that incorporate VDBP, multiple vitamin D metabolites, and other variables known to be associated with vitamin D, such as intact parathyroid hormone (iPTH), would more accurately predict measured free 25(OH)D concentrations compared with earlier equations.

## Methods

### Study population

The Health ABC study is an interdisciplinary, longitudinal study focused on risk factors for the decline of function in community‐dwelling older persons.^[^ ^20^ ^]^ Recruitment and data collection were performed on 3075 community‐dwelling 70‐ to 79‐year‐old adults from Memphis, TN, and Pittsburgh, PA, between 1997 and1998. Serum measurements for this analysis were performed in a random subcohort as part of a study evaluating kidney disease and other health outcomes, as described previously.^[^ ^6^ ^]^ The Health ABC study was approved by the local institutional review boards (IRBs) and all of the participants provided informed consent. The present study was approved by the IRB at the University of California, San Diego.

### Vitamin D measurements

Prior to blood sampling, participants were required to fast for ≥8 h. Blood samples were stored at −70°C from collection in 1998 to 1999 until testing. The predictor variables for this analysis included VDBP and the vitamin D metabolites 25(OH)D_3_, 25(OH)D_2_, 24,25(OH)_2_D_3_, 1,25(OH)_2_D_3_, and 1,25(OH)_2_D_2_. The vitamin D metabolites were quantified using immunoaffinity enrichment and liquid chromatography–tandem mass spectrometry.^[^ ^6^ ^]^ VDBP concentration and phenotype were determined simultaneously via liquid chromatography–tandem mass spectrometry, as described previously with minor assay modifications.^[^ ^21^ ^]^ Intra‐assay coefficients of variation range from 3.1% to 9.1% across an array of concentrations of VDBP. The vitamin D metabolite ratio (VMR) was included in this analysis and was calculated by dividing serum 24,25(OH)_2_D_3_ by serum 25(OH)D_3_ and then multiplying by 100.^[^ ^7^, ^11^ ^]^ As we found no spectrometric evidence of 24,25(OH)_2_D_2_, the VMR was calculated using 24,25(OH)_2_D_3_ and 25(OH)D_3_ only.

The measured outcome variable was free 25(OH)D, which includes both free 25(OH)D_2_ and free 25(OH)D_3_. Free 25(OH)D was quantified using the Diasource competitive ELISA according to the manufacturer's instructions. The intra‐assay coefficient of variation of the assay was 8.9% to 16.7%.

### Other measurements

Study participants provided a medical history and a physical examination. Participants self‐reported their demographics and smoking status. Weight was measured using a balance beam scale and height was measured using a Harpenden stadiometer (Holtain Ltd). Body mass index (BMI) was calculated in kg/m^2^. Baseline prevalent diabetes was defined using self‐reported histories, use of antidiabetic agents, fasting plasma glucose concentration >125 mg/dL, or a 2‐h oral glucose tolerance test result >199 mg/dL. Systolic and diastolic blood pressures were measured twice using a conventional mercury sphygmomanometer and averaged. Medications were brought into study visits by participants and categorized using the Iowa Drug Information System.

Serum cystatin C and urine albumin and urine creatinine measurements were available only at year 1 (baseline), so we carried forward these measurements to year 2 when vitamin D metabolites and all other data were collected, consistent with prior studies.^[^ ^6^, ^11^ ^]^ Urine albumin was measured using a particle‐enhanced turbidimetric inhibition immunoassay allowing for direct albumin quantification (Siemens). Measurement of urine creatinine was done by a modified Jaffé method on a clinical chemistry analyzer (Siemens). Cystatin C was measured at the Health ABC core laboratory (University of Vermont, Burlington, VT, USA) with a BNII nephelometer (Dade Behring Inc.) that used a particle‐enhanced immunonephelometric assay (N Latex Cystatin C). Estimated glomerular filtration rate (eGFR) was calculated using the 2012 CKD Epidemiology Collaboration (CKD‐EPI) cystatin C equation.^[^ ^22^ ^]^ Serum calcium and phosphate were measured using direct quantitative colorimetric determination (Stanbio Laboratory). Intact parathyroid hormone (iPTH) was measured in EDTA plasma using a two‐site immunoradiometric assay kit (N‐tact PTHSP; DiaSorin). Fibroblast growth factor‐23 (FGF‐23) was measured using an intact assay (Kainos Laboratories). Serum albumin was measured using the same assay for VDBP with the addition of internal standards for three albumin‐specific peptides, as described previously.^[^ ^11^ ^]^

### Statistical analysis

We used a randomly generated internal development data set of two‐thirds of the participants (*n* = 244) and least‐squares linear regression to develop estimating equations. Prior to inputting the variables into the linear regression models, all vitamin D metabolite measurements, as well as VDBP, iPTH, and intact fibroblast growth factor‐23 (iFGF‐23), were log transformed due to their known non‐normal distributions. We statistically assessed the participant characteristics associated with different free 25(OH)D levels and prioritized the significant results for use in the forward selection process when developing the equations. The models evaluated total 25(OH)D, 24,25(OH)_2_D_3_, 1,25(OH)_2_D_3_, VDBP concentration, VDBP phenotype, age, sex, race, BMI, season of measurement, clinical site, diabetes, eGFR (calculated using cystatin C), calcium, phosphate, iPTH, and iFGF23. To provide a parsimonious list of variables, only those that improved the adjusted *R* ^2^ by ≥0.02 were retained. After variables were added to the initial model, multiplicative interaction terms were generated by multiplying each of the retained variables with one another. Each interaction term was evaluated in the regression model and retained if it satisfied the criteria stated earlier. Three equations were created. The first equation forced 25(OH)D_2_, 25(OH)D_3_, VDBP, and serum albumin concentrations, given their known relationship with free 25(OH)D and their inclusion in the equation used by both current estimating equations.^[^ ^23^ ^]^ The second equation forced these same variables plus VDBP genetic phenotypes.^[^ ^24^, ^25^ ^]^ The third equation forced no variables but only included variables that are available at a typical clinical laboratory, such as 25(OH)D_3_, iPTH, serum albumin, and 1,25(OH)_2_D_3_.

The remaining 126 participants not randomly selected for estimating equation development were utilized for internal validation. We calculated estimated free 25(OH)D values for each participant using the three newly derived equations as well as the equation published by Bikle et al.^[^ ^23^ ^]^ We utilized Bland‐Altman plots to explore the mean difference between the estimated free 25(OH)D from each equation and measured 25(OH)D as a marker of bias. Equation precision was evaluated by the 95% limits of agreement (LOA) in Bland‐Altman plots. To assess overall accuracy, we calculated the percentage of persons among whom estimated free 25(OH)D by each equation was within 15% (P15) and 30% (P30) of measured free 25(OH)D. To compare the performance of our linear regression models, we compared the difference in root mean square error (RMSE) between the development and validation data. Analyses were conducted using Stata statistical software version 15.1 (Stata Corporation, College Station, TX, USA).

## Results

### Clinical characteristics

The mean age of the 370 individuals in the study was 75 ± 3 years; 53% were women, 40% were Black, and 24% had eGFR <60 mL/min/1.73 m^2^. The median [interquartile range (IQR)] total 25(OH)D_3_ concentration was 20.3 [13.4, 28.9] ng/mL, and total 25(OH)D_2_ was 0.33 [0.2, 1.2] ng/mL. The median [IQR] free 25(OH)D concentration was 5.3 [4.1 to 6.6] pg/mL and median VDBP concentration was 253.3 [226.9 to 284.1] ug/mL. The most prevalent VDBP phenotype was homozygous Gc1s (25%). The median [IQR] 24,25(OH)_2_D_3_, 1,25(OH)_2_D_3_, and VMR concentrations were 1.7 [0.9 to 2.9] ng/mL, 40.2 [30.2 to 50.4] ng/mL, and 8.9 [6.5 to 11.4] (ng/mL)/(ng/mL), respectively. Baseline characteristics across quartiles of free 25(OH)D are shown in Table 1. Compared to persons in the lowest free 25(OH)D quartile, those with higher free 25(OH)D concentrations were more often male and White, had lower BMIs, had lower VDBP concentrations, and were more likely to have chronic kidney disease (CKD). Persons with higher free 25(OH)D also had lower iPTH and higher iFGF23 concentrations. Free 25(OH)D concentrations were highest among persons with VDBP genotypes that were homozygous Gc1s and Gc2/Gc1s and lowest among those who were homozygous Gc2 and heterozygous Gc2/Gc1f. Free 25(OH)D concentrations were highest in the summer and lowest in the spring. Vitamin D supplementation was 1% in the lowest free 25(OH)D quartile and 9% to 15% in the other three quartiles.

**Table 1:** Baseline Characteristics of Health ABC Participants by Free 25(OH)D Quartiles

	Q1 (*n* = 93)	Q2 (*n* = 92)	Q3 (*n* = 93)	Q4 (*n* = 92)
Range (pg/mL)	1.59–4.08	4.10–5.25	5.28–6.63	6.64–10.5
Age (years) ± SD	74.2 (3.0)	74.7 (2.8)	74.6 (3)	75.0 (2.8)
Female, *n* (%)	60 (65)	49 (53)	45 (48)	42 (46)
White, *n* (%)	35 (38)	51 (55)	63 (68)	72 (78)
Clinic site, *n* (%)				
Memphis	46 (49)	60 (65)	39 (42)	45 (49)
Pittsburgh	47 (51)	32 (35)	54 (58)	47 (51)
Season of blood measurement, *n* (%)				
Winter	15 (16)	30 (33)	26 (28)	23 (25)
Spring	43 (46)	27 (29)	22 (24)	18 (20)
Summer	15 (16)	14 (15)	22 (24)	33 (36)
Fall	20 (22)	21 (23)	23 (25)	18 (20)
BMI (kg/m^2^) ± SD	28.3 (4.8)	27.9 (4.7)	27.1 (4.8)	25.7 (3.8)
Smoking status, *n* (%)				
Never	42 (45)	34 (37)	41 (44)	44 (48)
Former	41 (44)	49 (53)	45 (48)	44 (48)
Current	10 (11)	9 (10)	7 (8)	4 (4)
Diabetes, *n* (%)	41 (44)	43 (47)	37 (40)	33 (36)
Systolic BP (mm Hg) ± SD	137 (21)	137 (21)	129 (20)	137 (23)
Diastolic BP (mm Hg) ± SD	75 (12)	70 (11)	69 (10)	70 (11)
Anti‐HTN medications, *n* (%)	61 (66)	62 (67)	58 (63)	53 (58)
Vitamin D supplementation, *n* (%)	1 (1)	8 (9)	14 (15)	10 (11)
eGFR (mL/min/1.73m^2^) ± SD	74 (20)	73 (19)	72 (18)	68 (19)
Urine ACR, median [IQR]	7.8 [4.1–48.1]	9.5 [5.8–37.4]	7.3 [4.4–17.3]	10.4 [4.3–22.8]
Calcium (mg/dL) ± SD	8.9 (0.5)	8.9 (0.5)	8.9 (0.4)	8.9 (0.4)
Phosphate (mg/dL) ± SD	3.6 (0.5)	3.5 (0.5)	3.6 (0.5)	3.6 (0.5)
iPTH (pg/mL), median [IQR]	42.9 [30.5–60.3]	36.3 [27.0–46.8]	31.6 [23.5–41.5]	28.7 [21.1–38.6]
FGF23 (pg/mL, median [IQR]	43.8 [30.9–54.1]	44.4 [33.9–58.6]	39.6 [3.7–59.0]	49.1 [36.4–63.7]
VDBP Genetic Phenotype, *n* (%)				
Gc1f/Gc1f	26 (28)	23 (25)	14 (15)	9 (10)
Gc1f/Gc1s	23 (25)	8 (9)	13 (14)	16 (17)
Gc1s/Gc1s	15 (16)	27 (29)	17 (18)	32 (35)
Gc2/Gc1f	17 (18)	12 (13)	14 (15)	5 (5)
Gc2/Gc1s	9 (10)	18 (20)	26 (28)	25 (27)
Gc2/Gc2	3 (3)	4 (4)	9 (10)	5 (5)
Serum albumin (g/dL) ± SD	4.3 (0.4)	4.3 (0.4)	4.3 (0.4)	4.4 (0.4)
25(OH)D_3_ (ng/mL), median [IQR]	12.3 [8.8–16.4]	18.8 [12.8–23.9]	23.0 [17.1–31.2]	32.1 [23.1–37.4]
25(OH)D_2_ (ng/mL), median [IQR]	0.2 [0.2–0.4]	0.3 [0.2–0.8]	0.4 [0.2–2.4]	0.5 [0.2–5.0]
24,25(OH)_2_D_3_ (ng/mL), median [IQR]	0.7 [0.4–1.2]	1.6 [0.8–2.3]	2.2 [1.5–3.3]	3.4 [2.2–4.9]
1,25(OH)_2_D_3_ (pg/mL), median [IQR]	46 [37–57]	41 [32–51]	40 [30–49]	35 [27–44]
1,25(OH)_2_D_2_ (ng/mL), median [IQR]	0.3 [0.1–0.8]	0.3 [0.1–1.1]	0.3 [0.1–2.5]	0.2 [0.1–2.9]
VDBP (ug/mL), median [IQR]	261 [236–298]	256 [224–284]	239 [220–269]	260 [231–283]
VMR (ng/ml)/(ng/mL), median [IQR]	6.2 [4.6–8.3]	8.3 [5.8–10.5]	9.6 [7.5–12.0]	11.1 [9.0–14.1]

*Note*: Values for continuous variables given as mean ± SD or median [Q1, Q3]; values for categorical variables, as count (percentage).

Abbreviations: ACR, albumin‐creatinine ratio; BP, blood pressure; eGFR, estimated glomerular filtration rate (based on cystatin C); FGF‐23, fibroblast growth factor 23; HDL, high‐density lipoprotein; LDL, low‐density lipoprotein; iPTH, intact parathyroid hormone; VDBP, Vitamin D binding protein; VMR, vitamin D metabolite ratio.

### Equation development

We developed three equations to estimate free 25(OH)D (Table 2). The first equation forced 25(OH)D_3_, VDBP concentration, and serum albumin because these variables were used in the Bikle equation (*R* ^2^ = 0.499). The other variables that sufficiently improved the model to be retained included 25(OH)D_2_, 1,25(OH)_2_D_3_, 24,25(OH)_2_D_3_, and a multiplicative interaction term 25(OH)D_3_ × 24,25(OH)_2_D_3_. The model's final adjusted *R* ^2^ was 0.586. The second equation forced the same initial variables as Eq. (1) as well VDBP phenotype. The other variables included were 25(OH)D_2_, 1,25(OH)_2_D_3_, 24,25(OH)_2_D_3_, and interaction term 25(OH)D_3_ × 24,25(OH)_2_D_3_ with an adjusted *R* ^2^ of 0.583. Our third equation did not initially force any variables but only tested variables for inclusion that are available at a typical clinical laboratory. It ultimately retained 25(OH)D_3_, serum albumin, iPTH, and 1,25(OH)_2_D_3_. No interaction terms improved the model fit, and the model's final adjusted *R* ^2^ was 0.514, which is lower than that observed in Eqs. (1) and (2); however, this *R* ^2^ remained slightly higher than that of the Bikle equation in our data set (*R* ^2^ = 0.499).

**Table 2:** Final Free 25(OH)D Estimation Equations

	Adjusted *R* ^2^	RMSE (development, validation)
Eq. (1): Albumin^a^, 25(OH)D_3_ ^a^, 25(OH)D_2_ ^a^, VDBP^a^, 1,25(OH)_2_D_3_, 24,25(OH)_2_D_3_		
[Free 25(OH)D] = 12.202 + 0.359 × albumin + 0.844 × log[25(OH)D_3_] + 0.145 × log[25(OH)D_2_] − 1.522 × log(VDBP) − 0.851 × log[1,25(OH)_2_D_3_] − 1.906 × log[24,25(OH)_2_D_3_] + 0.943 × log[25(OH)D_3_]^b^ × log[24,25(OH)_2_D_3_]	0.586	1.159, 1.161
Eq. (2): albumin, 25(OH)D_3_ ^a^, VDBP^a^, 25(OH)D_2_ ^a^, VDBP phenotype^a^, 1,25(OH)_2_D_3_, 24,25(OH)_2_D_3_		
[Free 25(OH)D] = 12.779 + 0.348 × albumin + 0.748 × log[25(OH)D_3_] − 1.566 × log(VDBP) + 0.149 × log[25(OH)D_2_] − 0.833 × log[1,25(OH)_2_D_3_] − 1.826 × log[24,25(OH)_2_D_3_] + 0.935 × log[25(OH)D_3_]^b^ × log[24,25(OH)_2_D_3_] [+0 if VDBP Phenotype is Gc1f/Gc1f], [+0.019716 if Phenotype Gc1f/Gc1s], [+0.002741 if Phenotype Gc1s/Gc1s], [−0.394562 if Phenotype Gc2/Gc1f], [−0.1283514 if Phenotype Gc2/Gc1s], [−0.1381116 if Phenotype Gc2/Gc2]	0.583	1.164, 1.165
Eq. (3): albumin, 25(OH)D_3_, iPTH, 1,25(OH)_2_D_3_		
[Free 25(OH)D] = 3.098 + 0.275 × albumin +2.370 × log[25(OH)D_3_] − 0.467 × log(iPTH) − 1.194 × log[1,25(OH)_2_D_3_]	0.514	1.257, 1.353
Bikle equation: 25(OH)D, VDBP, albumin [Free 25(OH)D] = Total 25(OH)D/(1 + K_a1_ × VDBP + K_a2_ × albumin)	0.499	

*Note*: *K_an_ is affinity constant of protein for 25(OH)D: K_a1_ = 8.00 × 10^8^ M^−1^, K_a2_ = 6.00 × 10^5^ M^−1^.

^a^ Variables forced into the equation.

^b^ Units: albumin (g/dL), 25(OH)D_3_ (ng/mL), VDBP (ug/mL), 25(OH)D_2_ (ng/mL), 1,25(OH)_2_D_3_ (pg/mL), 24,25(OH)_2_D_3_ (ng/mL), iPTH (pg/mL), total 25(OH)D (ng/mL).

[Correction added on 24 August 2023, after first online publication: Interaction term 25(OH)D_3_^b^ 24,25(OH)_2_D_3_ has been changed to logarithmic forms of both terms log[25(OH)D_3_] × log[24,25(OH)_2_D_3_]]

### Internal validation

**Fig. 1:**
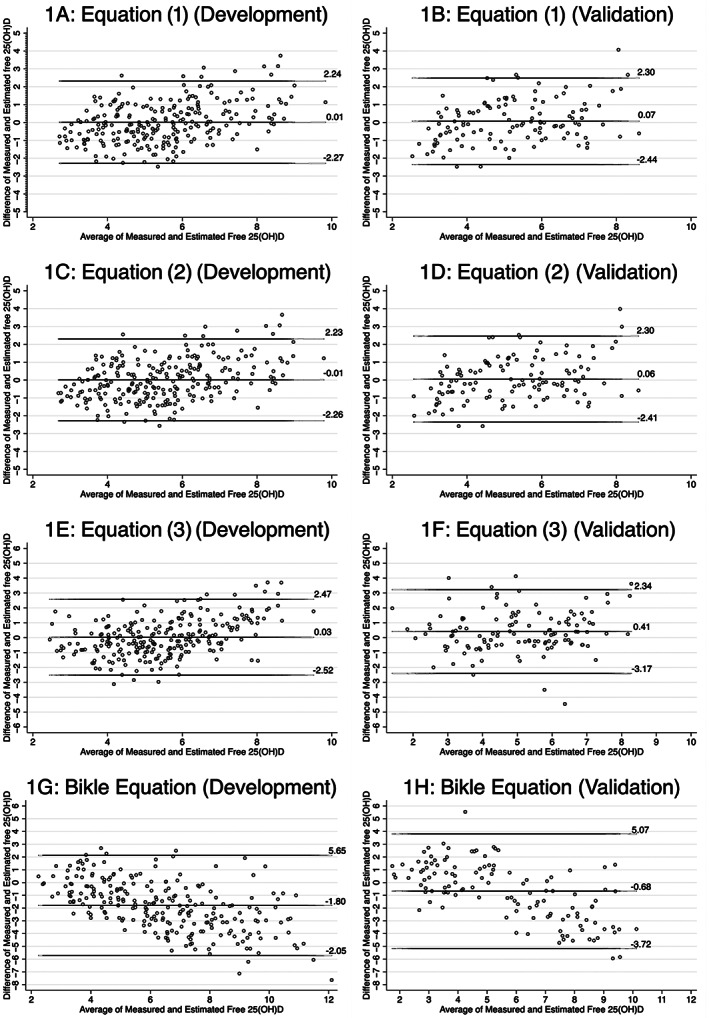
(A–H) Bland‐Altman plots of measured and calculated free 25(OH)D in development and validation data sets for new equations and Bikle equation. The Bland‐Altman plots illustrate the mean bias and 95% LOAs for all three newly developed equations, as well as the existing equation by Bikle, in both the development and validation data sets.

We evaluated Bland‐Altman plots (Fig. 1A–H) to assess bias with each equation. Equations (1) and (2) showed minimal bias in the validation plots (0.07 [95% LOA: −2.44, 2.30] pg/mL and 0.06 [95% LOA: −2.41, 2.30] pg/mL) using Eqs. (1) and (2), respectively (Table 3). Additionally, there was greater bias in participants who were men, Black, or with no history of CKD in Eqs. (1) and (2) (Table S1A–C). We observed a systematic bias in Eq. (3)'s validation plot (0.41 [95% LOA: −3.17, 2.34] pg/mL), which results in an overestimation of calculated free 25(OH)D compared to measured 25(OH)D. There was higher bias in participants without CKD and in Black participants for Eq. (3). The opposite direction of bias was observed in Bland‐Altman plots of the Bikle estimating equation, which showed a systematic underestimation in the overall sample [−1.42 (95% LOA: −2.74, 5.59) pg/mL], but this differed by the level of free 25(OH)D, as they were systematically underestimated at lower concentrations and overestimated at higher concentrations, and the 95% LOAs were wider compared to the other three equations.

**Table 3:** Evaluation of Equations within Development and Validation Data Sets

Measured free 25(OH)D (pg/mL) (mean ± SD)	5.37 +/− 1.81 Development data set (244)	Validation data set (126)	Total cohort (370)
Eq. (1): albumin, 25(OH)D_3_, 25(OH)D_2_, VDBP, 1,25(OH)_2_D_3_, 24,25(OH)_2_D_3_			
Proportion within 15%	49%	45%	
Proportion within 30%	77%	79%	
Bias from measured free 25(OH)D mean (pg/mL) (95% LOA)	0.01 (−2.27, 2.24)	0.07 (−2.44, 2.30)	
Eq. (2): albumin, 25(OH)D_3_, VDBP, 25(OH)D_2_, VDBP phenotype, 1,25(OH)_2_D_3_, 24,25(OH)_2_D_3_			
Proportion within 15%	48%	45%	
Proportion within 30%	79%	79%	
Bias (pg/mL) (95% LOA)	−0.01 (−2.26, 2.23)	0.06 (−2.41, 2.30)	
Eq. (3): albumin, 25(OH)D_3_, iPTH, 1,25(OH)_2_D_3_			
Proportion within 15%	47%	46%	
Proportion within 30%	78%	75%	
Bias (pg/mL) (95% LOA)	0.03 (−2.52, 2.47)	0.41 (−3.17, 2.34)	
Bikle equation: 25(OH)D, VDBP, albumin			
Proportion within 15%	17%	10%	22%
Proportion within 30%	42%	26%	43%
Bias (pg/mL) (95% LOA)	−1.80 (−2.05, 5.65)	−0.68 (−3.72, 5.07)	−1.42 (−2.74, 5.59)

Table 3 also shows the accuracy of the newly developed equations as well as the Bikle equation in the development and internal validation data sets.^[^ ^23^ ^]^ In the validation data sets, the estimated free 25(OH)D in Eqs. (1), (2), and (3)was within 15% of the measured free 25(OH)D (P_15_) 45%, 45%, and 46%, respectively. The estimations were within 30% (P_30_) of measured free 25(OH)D 79%, 79%, and 75%, for Eqs. (1), (2), and (3), respectively. In the entire cohort, Bikle's equation's P15 and P30 were 22% and 43%, respectively. The difference in RMSE between the validation and development groups for Eqs. (1), (2), and (3) was 0.002, 0.001, and 0.096, respectively. Table S1A–C indicated that the new equations performed more accurately in P30 in participants who were men, White, or without CKD.

## Discussion

We developed and internally validated three new equations to estimate free 25(OH)D in a multiracial cohort of community‐living older men and women. All three equations included albumin, 25(OH)D_3_, and 1,25(OH)_2_D_3_ but differed in their inclusion of other vitamin D metabolites. All of these equations had moderate accuracy. We also observed that a prior equation published by Bikle and colleagues appeared to have systematic bias, wherein it underestimated free 25(OH)D concentrations at lower levels and overestimated them at higher levels. Each of the new equations appeared to have lower bias and higher accuracy than the currently used equation. However, it should be noted that our equations were developed and validated internally, whereas the previous equation was externally validated here. To our knowledge, this is also the first study to evaluate incorporation of VDBP phenotype data into a free 25(OH)D estimating equation; however, we demonstrated that its inclusion did not meaningfully improve estimation of free 25(OH)D concentrations.

The two primary free 25(OH)D estimating equations used in other studies include Bikle's equation as well as a modified Vermulean equation.^[^ ^12^, ^13^, ^14^, ^15^ ^]^ Bikle's free 25(OH)D estimating equation was developed using 64 subjects (22 healthy and 42 with liver disease) and included total 25(OH)D, serum albumin, VDBP, and an affinity constant of protein binding for albumin and VDBP, but it did not evaluate VDBP genetic phenotypes.^[^ ^15^ ^]^ Additionally, the prior equation demonstrated relationships of measured free 25(OH)D concentrations with iPTH and calcium concentrations, but these variables were not included in the estimating equation.^[^ ^15^ ^]^ We evaluated iPTH in our equation development and found that it was important enough for free 25(OH)D estimation to be retained in our “clinical model” in Eq. (3).

The equation used to estimate free testosterone was developed by Vermulean et al. and was subsequently modified to estimate free 25(OH)D. This equation does not include VDBP phenotypes or iPTH.^[^ ^14^ ^]^ We chose to compare our new equations to Bikle's equation because it was more widely used in prior studies.^[^ ^16^ ^]^ Several studies investigated the relation between directly measured free 25(OH)D and calculated free 25(OH)D using the two previously mentioned equations.^[^ ^12^, ^16^, ^17^, ^18^ ^]^ Schwartz et al. found that calculated free 25(OH)D was positively correlated with measured free 25(OH)D but explained only 13% of its variability using the equation developed by Bikle.^[^ ^16^ ^]^ Other studies showed that the correlations ranged from *r* = 0.4 to *r* = 0.8, and the average estimated values of free 25(OH)D using these equations were typically higher compared to direct measurements.^[^ ^12^, ^16^, ^17^, ^18^ ^]^ Thus, improved free 25(OH)D equations may be beneficial for future studies to evaluate the associations of free 25(OH)D with clinical outcomes or with other biomarkers where free 25(OH)D measurements are not available. Here, we provided three new free 25(OH)D equations that appeared to better correlate with measured free 25(OH)D than the current equations in community‐dwelling older adults.

Our initial hypothesis was that the incorporation of VDBP and VDBP phenotype into a free 25(OH)D equation would significantly improve the performance of estimating equations because we believed both the VDBP concentration itself, and its genetic phenotypes which influence binding affinities were likely to influence free 25(OH)D concentrations. The equations, which included VDBP with or without VDBP phenotypes, had improved correlation (Eq. 1: *r* = 0.586 and Eq. 2: *r* = 0.583) compared to the equation without VDBP or its phenotypes (Eq. 3: *r* = 0.514); however, the P15 and P30 were similar among all three equations. We also note that an equation that included VDBP phenotype (Eq. 2) versus one without it (Eq. 1) performed nearly identically (Eq. 2: *r* ^2^ = 0.583 and Eq. 1: *r* ^2^ = 0.586). Therefore, the incorporation of VDBP phenotype (Eq. 2) may not be critical for estimating free 25(OH)D concentrations.

It is interesting to note that the coefficients in Eq. (2) do not perfectly align with the reported binding affinity hierarchy for VDBP genotypes and vitamin D metabolites (Gc1f > Gc1s > Gc2).^[^ ^24^ ^]^ However, this discrepancy could be due to the limitations of our sample size or the complex relationships of calcium, phosphorus, and PTH on free vitamin D serum concentrations in different individuals.

On analysis of the Bland‐Altman plots, the overall accuracy of Eq. (3) is similar to that of Eqs. (1) and (2); however, there is larger bias with this equation than the other two. The Bland‐Altman plot of the Bikle estimating equation shows an underestimation in the study sample overall (−1.42). More importantly, this bias differed systematically by the level of measured free 25(OH)D, underestimating at lower levels and overestimating at higher levels. The 95% LOA are also wider for the Bikle equation compared to the other three equations. It is again vital to note that the estimating equation developed by Bikle was developed in a smaller, different participant population and is being externally validated here, whereas the new equations are being internally validated. Nonetheless, it appeared more biased and less precise and had lower overall accuracy than the three newly developed free 25(OH)D estimating equations.

The strengths of this study include its larger sample compared to previous studies that directly measured free 25(OH)D, its inclusion of both men and women and Black and White participants, and the availability of vitamin D metabolite measurements. This study also has important limitations. While the sample size was relatively large compared to prior studies, an even larger sample size would allow for more precision in within‐subgroup evaluations. Additionally, external validation outside of the Health ABC study will be needed to assure that these new equations perform similarly in other settings. The age range of participants in Health ABC is 70 to 79 years old, which may not be generalizable to younger individuals. Another limitation is that the intra‐assay coefficient of variation of the free 25(OH)D assay ranges above the ideal level of less than 10% (8.9%–16.7%). Finally, two of the equations require inputs that are not readily clinically available (Eqs. 1 and 2), so they would be difficult to use in standard practice, although they could be readily used for research purposes. For this reason, we developed a clinical equation (Eq. 3) which appeared to fit less well in model development but proved to perform similarly to the more expansive equations in our internal validation studies.

In conclusion, free 25(OH)D equations that include VDBP, with or without VDBP phenotypes, or clinically available laboratory measurements perform with little bias and moderate overall accuracy for estimating free 25(OH)D concentrations among community‐living older adults. A prior estimating equation proposed by Bikle et al., which was externally validated in our study, was found to have lower overall accuracy and a systematic bias depending on the concentration of measured free 25(OH)D, which was not evident using the three newly derived estimating equations. Future studies are required to externally validate these free 25(OH)D equations in different settings and to evaluate the relationship of measured and estimated free 25(OH)D with clinical outcomes such as cardiovascular disease, fractures, and mortality.

## Author Contributions

**Jonathan H. Cheng:** Conceptualization; formal analysis; investigation; methodology; writing – original draft; writing – review and editing. **Andrew Norbert Hoofnagle:** Data curation; resources; writing – review and editing. **Ronit Katz:** Formal analysis; methodology; writing – review and editing. **Stephen Kritchevsky:** Conceptualization; writing – review and editing. **Michael G. Shlipak:** Conceptualization; validation; writing – review and editing. **Mark Sarnak:** Conceptualization; writing – review and editing. **Joe Ix:** Conceptualization; methodology; supervision; writing – review and editing. **Charles Ginsberg:** Conceptualization; data curation; investigation; methodology; resources; supervision; writing – review and editing.

## Funding Information

This study was supported by grants from the National Institute on Aging T32: Improving the Health of Aging Women and Men Training Grant AG058529 and National Institutes of Health (NIH) Loan Repayment Grant (Dr. Cheng), NIH K23DK118197 (Dr. Ginsberg), National Institute of Diabetes, Digestive, and Kidney Diseases R01DK101720 and K24 DK110427 (Dr. Ix), National Institute on Aging 5R01AG027002 (Drs. Sarnak and Shlipak), and University of Washington Nutrition and Obesity Research Center P30DK035816 (Dr. Hoofnagle). The funders did not have a role in the study design, data collection, analysis, reporting, or the decision to submit for publication.

## Disclosures

The authors certify that they have no affiliations with or involvement in any organization or entity with any financial interest (such as honoraria; educational grants; participation in speakers' bureaus; membership, employment, consultancies, stock ownership, or other equity interest; and expert testimony or patent‐licensing arrangements) or nonfinancial interest (such as personal or professional relationships, affiliations, knowledge or beliefs) in the subject matter or materials discussed in this manuscript.

### Peer Review

The peer review history for this article is available at https://www.webofscience.com/api/gateway/wos/peer-review/10.1002/jbm4.10781.

## Supporting information


**Data S1.** Supporting Information.
