# Supplementary material for: Development and Validation of Novel Free Vitamin D Equations: The Health Aging and Body Composition Study
Source: JBMR Plus. 2023 Jun 13;7(9):e10781. doi: 10.1002/jbm4.10781 (PMC10494503; doi:10.1002/jbm4.10781)
Supplement: Supplementary file 1 — Data S1. Supporting Information. [file JBM4-7-e10781-s001.pdf]

Supplemental Table 1A: Evaluation of Equations within Development and Validation Data Sets Separated by Gender

Measured Free 25(OH)D (pg/ml) (Mean ± SD) 5.37 +/- 1.81

	Development Data Set (128)	Validation Data Set (68)	Total Cohort (196)	Development Data Set (121)	Validation Data Set (53)	Total Cohort (174)
**Gender**	**Women**			**Men**		
Equation 1						
Proportion within 15%	45%	38%		52%	55%	
Proportion within 30%	73%	75%		83%	85%	
Bias from measured free 25(OH)D mean (pg/ml) (95% LOA)	0.02 (-2.30, 2.34)	0.01 (-2.45, 2.47)		0.01 (-2.19, 2.20)	0.15 (-2.11, 2.41)	
Equation 2						
Proportion within 15%	42%	38%		55%	55%	
Proportion within 30%	75%	75%		83%	85%	
Bias from measured free 25(OH)D mean (pg/ml) (95% LOA)	0.02 (-2.29, 2.32)	0.003 (-2.44, 2.45)		0.01 (-2.18, 2.19)	0.12 (-2.14, 2.38)	
Equation 3						
Proportion within 15%	42%	43%		50%	55%	
Proportion within 30%	76%	68%		79%	87%	
Bias from measured free 25(OH)D mean (pg/ml) (95% LOA)	-0.02 (-2.49, 2.45)	0.42 (-2.46, 3.30)		0.08 (-2.44, 2.60)	0.40 (-2.21, 3.01)	
Bikle Equation						
Proportion within 15%	25%	25%	25%	18%	19%	18%
Proportion within 30%	38%	53%	43%	43%	43%	43%
Bias from measured free 25(OH)D mean (pg/ml) (95% LOA)	-1.53 (-5.22, 2.16)	-0.46 (-4.68, 3.76)	-1.15 (-5.15, 2.85)	-2.08 (-6.02, 1.87)	-0.95 (-5.56, 3.65)	-1.73 (-6.00, 2.54)

Supplemental Table 1B: Evaluation of Equations within Development and Validation Data Sets Separated by Race

Measured Free 25(OH)D (pg/ml) (Mean ± SD)	5.37 +/- 1.81					
**Race**	Development Data Set (104)	Validation Data Set (45)	Total Cohort (149)	Development Data Set (145)	Validation Data Set (76)	Total Cohort (221)
	**Black**			**White**		
Equation 1						
Proportion within 15%	48%	36%		49%	51%	
Proportion within 30%	77%	73%		78%	83%	
Bias from measured free 25(OH)D mean (pg/ml) (95% LOA)	0.15 (-2.04, 2.33)	0.21 (-2.20, 2.62)		-0.09 (-2.38, 2.21)	-0.01 (-2.36, 2.33)	
Equation 2						
Proportion within 15%	46%	33%		50%	53%	
Proportion within 30%	78%	71%		79%	84%	
Bias from measured free 25(OH)D mean (pg/ml) (95% LOA)	0.16 (-2.02, 2.33)	0.17 (-2.26, 2.60)		-0.09 (-2.37, 2.19)	-0.01 (-2.33, 2.31)	
Equation 3						
Proportion within 15%	43%	24%		48%	62%	
Proportion within 30%	81%	62%		74%	84%	
Bias from measured free 25(OH)D mean (pg/ml) (95% LOA)	0.16 (-2.15, 2.46)	0.54 (-2.68, 3.75)		-0.07 (-2.68, 2.55)	0.34 (-2.11, 2.79)	
Bikle Equation						
Proportion within 15%	30%	24%	28%	16%	21%	18%
Proportion within 30%	53%	56%	54%	32%	45%	36%
Bias from measured free 25(OH)D mean (pg/ml) (95% LOA)	-1.10 (-4.74, 2.54)	0.35 (-3.26, 3.96)	-0.66 (-4.50, 3.19)	-2.31 (-6.00, 1.39)	-1.28 (-5.69, 3.12)	-1.95 (-6.01, 2.11)

Supplemental Table 1C: Evaluation of Equations within Development and Validation Data Sets Separated by CKD Status

Measured Free 25(OH)D (pg/ml) (Mean ± SD) 5.37 +/- 1.81						
**CKD Status**	Development Data Set (194)	Validation Data Set (87)	Total Cohort (281)	Development Data Set (55)	Validation Data Set (34)	Total Cohort (89)
	**No CKD**			**With CKD**		
Equation 1						
Proportion within 15%	48%	45%		51%	47%	
Proportion within 30%	76%	82%		82%	74%	
Bias from measured free 25(OH)D mean (pg/ml) (95% LOA)	0.04 (-2.28, 2.36)	0.10 (-2.17, 2.37)		-0.08 (-2.13, 1.96)	0.001 (-2.64, 2.64)	
Equation 2						
Proportion within 15%	46%	44%		55%	50%	
Proportion within 30%	78%	82%		82%	74%	
Bias from measured free 25(OH)D mean (pg/ml) (95% LOA)	0.03 (-2.28, 2.35)	0.08 (-2.18, 2.34)		-0.06 (-2.07, 1.95)	0.003 (-2.62, 2.63)	
Equation 3						
Proportion within 15%	43%	46%		56%	53%	
Proportion within 30%	75%	77%		84%	74%	
Bias from measured free 25(OH)D mean (pg/ml) (95% LOA)	0.11 (-2.46, 2.69)	0.49 (-2.01, 2.98)		-0.27 (-2.39, 1.85)	0.22 (-3.12, 3.57)	
Bikle Equation						
Proportion within 15%	23%	24%	23%	16%	18%	17%
Proportion within 30%	42%	51%	45%	35%	44%	38%
Bias from measured free 25(OH)D mean (pg/ml) (95% LOA)	-1.66 (-5.45, 2.13)	-0.56 (-4.75, 3.64)	-1.31 (-5.35, 2.73)	-2.28 (-6.20, 1.65)	-0.98 (-5.88, 3.92)	-1.78 (-6.25, 2.69)
